# Peptidomic Approaches and Observations in Neurodegenerative Diseases

**DOI:** 10.3390/ijms23137332

**Published:** 2022-06-30

**Authors:** Besnik Muqaku, Patrick Oeckl

**Affiliations:** 1 German Center for Neurodegenerative Diseases (DZNE e.V.), 89081 Ulm, Germany; besnik.muqaku@dzne.de; 2 Department of Neurology, Ulm University Hospital, 89081 Ulm, Germany

**Keywords:** neurodegenerative diseases, peptidomics, cerebrospinal fluid, mass spectrometry

## Abstract

Mass spectrometry (MS), with its immense technological developments over the last two decades, has emerged as an unavoidable technique in analyzing biomolecules such as proteins and peptides. Its multiplexing capability and explorative approach make it a valuable tool for analyzing complex clinical samples concerning biomarker research and investigating pathophysiological mechanisms. Peptides regulate various biological processes, and several of them play a critical role in many disease-related pathological conditions. One important example in neurodegenerative diseases is the accumulation of amyloid-beta peptides (Aβ) in the brain of Alzheimer’s disease (AD) patients. When investigating brain function and brain-related pathologies, such as neurodegenerative diseases, cerebrospinal fluid (CSF) represents the most suitable sample because of its direct contact with the brain. In this review, we evaluate publications applying peptidomics analysis to CSF samples, focusing on neurodegenerative diseases. We describe the methodology of peptidomics analysis and give an overview of the achievements of CSF peptidomics over the years. Finally, publications reporting peptides regulated in AD are discussed.

## 1. Introduction

Peptides have been the object of investigation for more than 100 years [1]. Nevertheless, peptidomics aiming at the identification and quantification of all peptides present in a biological sample is relatively new. Peptides play a decisive role in a large variety of biological processes. Peptide hormone secretin, the first discovered peptide, regulates fluid homeostasis in many organs and the pH value in the pancreas [2,3,4,5]. Other peptides act as anti-oxidants, such as carnosine, which plays an essential role in muscle [6], or some growth-factor-derived peptides show anti-microbial properties, which play a pivotal role in defense mechanisms [7]. Peptides initiate the killing of cancer cells by activating immune cells mediated by antigen-presenting cells [8]. Additionally, peptides are valuable diagnostic tools or therapeutic agents, such as hepcidin, which is a promising biomarker for iron deficiency diseases and insulin widely applied in diabetes treatment [9,10].

Neuropeptides are the most studied peptides. They are the largest signaling molecules in the nervous system and, by modulating the critical processes in the brain, influence the processes in the entire organism [11]. Neuropeptides are synthesized in an inactive form and only after several enzymatic processing steps does a mature state arise. The way to an active form might go through peptidases by removing the signal peptide, thus convertases cleaving specifically at dibasic amino acids, arginine and lysine, and carboxypeptidase [11,12]. Orexins (orexin-A and orexin-B) are hypothalamic neuropeptides that mediate communication between neurons by activating orexin receptors and inducing the increase in the intracellular calcium concentration in neurons. They play a vital role in maintaining wakefulness and the sleep-wake cycle [13,14]. The opioid neuropeptides dynorphins, among others, regulate pain-related processes, motor behavior, and negative emotional states [15,16,17]. Dynorphins are prodynorphin-derived peptides produced after enzymatic cleavage and perform their function by acting on opioid receptors [17]. Another well-known neuropeptide is somatostatin (SST), with the isoform SST-14 predominantly spread in the central nervous system and SST-28 in peripheral body organs as the pancreas and gut [18]. SST plays a critical role in modulating cognitive function, which might explain its down-regulation in patients with many neurodegenerative diseases like Parkinson’s disease, and Alzheimer’s disease (AD) [18]. The deposition of amyloid-beta (Aβ) peptides has been correlated with STT deficiency in an AD transgenic model [19]. The administration of the SST analog improved the memory of AD patients and this probably occured by increasing the activity of neprilysin, an enzyme that promotes the degradation of deposited Aβ peptides [20,21]. Aβ peptides, the critical players in the pathology of AD, are generated upon cleavage of amyloid precursor protein (APP) mainly by secretases α, β, and γ [22,23]. There are over 560 genes coding proteases describing the diversity of peptidome and 150 genes code protease inhibitors, demonstrating tight regulation of the synthesis of bioactive peptides, but as well as their decisive role in regulating biological processes [24]. Additionally, peptides can also be generated as a degradation product of proteins [24]. The small reading frames code the sequence of small peptides up to 100 amino acids, leading to the synthesis of the short open reading frame (sORF)-encoded polypeptides (SEPs) [25]. The peptide diversity expands further by post-translational modifications (PTMs) like C-terminal amidation, N-terminal acetylation, N-terminal pyroglutamation, phosphorylation, and glycosylation [26]. These examples illustrate the importance and complexity of peptides in physiological and pathophysiological processes of the brain.

Cerebrospinal fluid (CSF) is in direct contact with the brain and makes it a collector of many biomolecules. Changes in CSF composition might mirror the perturbation in brain-related physiological processes, for instance, as a result of a pathological condition. The CSF peptidome did not show substantial overlap with plasma or serum peptidome depicting its unique composition and regulates processes related to protein activation cascade, blood coagulation, complement activation, regulation of axogenesis, and macromolecular complex remodeling [27]. Therefore, peptidomics, with the ability to measure thousands of peptides in CSF, depict a valuable tool in biomarker research, investigating the pathophysiological mechanisms and monitoring of the treatment response, all addressing brain functions and neurodegenerative diseases.

In this review, we evaluate studies applying the peptidomics approach to analyze CSF in neurodegenerative diseases. We provide an overview of the analytical steps of CSF peptidomics analysis and a chronological recapitulation of the CSF peptidome regarding neurodegenerative diseases. Reports on potential biomarkers of AD are discussed in detail, including those analyzing the plasma and serum.

## 2. Methods

In the PubMed database, we searched combinations of the following terms: peptidomics or peptidome, cerebrospinal fluid or CSF, and neurodegenerative diseases. In the next step, we searched for specific neurodegenerative diseases separately. Neurodegenerative diseases included in the search were as follows: Alzheimer’s disease, frontotemporal dementia, Lewy body dementia, Huntington’s disease, amyotrophic lateral sclerosis (ALS), Parkinson’s disease, Creutzfeldt-Jakob disease, frontotemporal lobar degeneration (FTDL), multiple system atrophy, prion diseases, Batten disease, and spinal muscular atrophy. Any search combination contained at least either the term cerebrospinal fluid or peptidomics. By replacing the term cerebrospinal fluid with plasma or serum, only a few additional entries were added to the existing list; therefore, they were included and discussed in the AD section. Based on these criteria, 82 publications were identified.

As we focused on neurodegenerative diseases and in experiments with CSF samples, in the next step we excluded publications containing the following terms in their title or abstract: cancer, cardiac arrest, liver, lung, heart, tissue, brain, culture, or cell. We were predominantly interested in works analyzing peptidome; therefore, publications reporting the measurement of a single or only a few peptides in their title or abstact were additionally excluded from further evaluation. The exclusion criteria were applied directly on PubMed and titles and abstracts were also manually inspected. To this end, publications reporting work on proteomics, inflammatory diseases, workshops, and conference articles were removed as well. The number of entries was reduced to 63.

A deeper evaluation of the remaining articles by applying the above criteria reduced the number of articles to 45. From them, 21 reviews and two book chapters were excluded. Two of the remaining publications analyzed the serum samples and another one plasma.

## 3. Methods Used for the Peptidomic Analysis of Biofluids

Although there are antibody-based assays measuring selected peptides [28], nowadays, peptidomics analysis is inseparably linked to mass spectrometry (MS). The entire MS-based analysis might take up to several days and is usually divided into three central parts: sample preparation, sample measurement, and data analysis [29]. Any of these steps can be prone to variations that might dictate even the final result of an entire study. Notably, close attention should be dedicated to the analysis of biofluids, such as CSF, plasma or serum, as these are the most used samples in biomarker research and are usually accompanied by measurement of up to several hundred samples [30,31,32]. This section addresses the analytical aspects in the peptidomics analysis of CSF samples.

The complexity of clinical samples containing several hundred and thousand biomolecules at a concentration range of up to 10 orders of magnitude represents a massive challenge in their analysis [33]. Although the protein concentration in CSF samples (200–700 µg/mL) compared with the plasma and serum (70–80 mg/mL) is much lower, it still remains a complex sample. A high albumin and immunoglobulins content (up to 70% of total protein amount in CSF) and a wide dynamic range of protein concentration challenge the determination of the regulatory biomolecules typically present at low concentrations [34]. In peptidomics analysis, the removal of other low molecular weight or small molecules is based on the hydrophobic properties of peptides and usually is done with desalting C18 columns prior to LC-MS measurement or directly in pre-column of chromatographic systems. The separation of peptides from proteins is a must in peptidomics analysis. For this, three main strategies are usually applied: protein precipitation with organic solvents, acidic protein precipitation, and ultrafiltration using a molecular weight cut-off (MWCO) filter [35,36,37,38]. However, many peptides and those building complexes with proteins will co-precipitate, thus reducing the identification rate of peptides [36]. Therefore, filters with different cut-off sizes are the most followed strategy for peptide separation from proteins [38]. Frequently, samples are supplemented with chaotropic reagents prior to filtration, such as guanidine hydrochloride to interrupt complexes with proteins, or the disulfide bonds were reduced by treatment with reducing reagents [35,39]. There are different reports regarding the appropriate cut-off size for the peptidome experiment and 30 kDa MWCO filters are the most used ones. Another strategy lately spread among the proteomics community is bead-based protein precipitation and digestion, the single-pot, solid phase-enhanced sample-preparation (SP3) [40]. Indeed, years ago, the strategy of using magnetic beads for peptide capture in CSF samples was reported [41,42].

Technological progress in MS instrumentations and particularly the increased resolution power empowered scientists to use MS to characterize intact proteins with a noted interest in analyzing antibodies. However, in conventional proteomics experiments, proteins prior to LC-MS analysis are digested to lower molecular weight molecules, peptides [30,31]. Hence, with respect to LC-MS measurements, both fields, proteomics and peptidomics, are identical, which enabled peptidome analysis to make use of technological and methodological development from the proteomics community. The application of electrospray ionization and matrix-assisted laser desorption/ionization (MALDI) as ionization techniques is a common practice in peptidomics, with the first one being widely spread due to the technical possibility of online coupling with chromatographic systems for peptide separation [36,38,41,42,43,44,45]. Almost all types of MS instruments, e.g., time-of-flight (TOF), linear ion trap, and Orbitrap, have been used in peptidome experiments, although, in the last years, the advantages of the usage of an Orbitrap hybrid instrument are more visible [39,46].

An untargeted approach with its explorative character aims to screen for all possible peptides in a clinical sample. In a data-dependent analysis (DDA) after a survey scan of unfragmented ions, top intense m/z peaks are selected for separation and consecutive isolation, followed by ion fragmentation and the acquisition of MS/MS spectra [47]. Because of the high complexity of clinical samples and despite chromatographic pre-separation and high-resolution MS spectra of unfragmented ions, ion fragmentation and the acquisition of MS/MS spectra are required for reliable identification. In the end, the MS/MS spectra linked to parent ion with the support of search engines integrated into software packages and by comparison with in-silico spectra collected in databases are assigned to peptides leading to their identification [48,49,50,51,52,53].

MS-based analysis generates a vast amount of very complex data. Consequently, software packages are needed for high-throughput data analysis [48,49,54]. The majority of them use databases containing all possible theoretical spectra and identification is based on matching with experimental MS/MS data. Even though isolated peptides might be further digested with trypsin in a few peptidomics experiments, such as in proteomics analysis, this is an exception rather than a standard procedure. This has enormous implications in data analysis. Trypsin predominantly cleaves proteins at the C-terminal side of the amino acid arginine and lysine, producing peptides with a sequence proceeded or followed by these two amino acid residues. In this case, the search engine algorithm focuses the search only on tryptic peptides. When no enzyme or unspecific is chosen as the digestion mode in software, such as in peptidomics, the software search space increases dramatically, and, with that, the search time increases up to 10-fold.

## 4. History and Advancements in CSF Peptidome

A two-phase extraction system of organic solvents was used for peptide isolation in one of the first peptidomics screening experiments aiming to characterize the CSF peptidome by means of MS. Prior to MS analysis, peptides were fractionated by liquid chromatography. In total, eight peptides and four peptide fragments were identified: fibrinopeptide A, C4A anaphylatoxin; N-terminal fragments of neuroendocrine specific protein VGF, ribonuclease 1; internal fragments of endothelin-binding receptor-like protein 2, chromogranin B/secretogranin I, chromogranin A, proenkephalin; and C-terminal fragments of neuroendocrine protein 7B2, pro-IGF-II, testican, and osteopontin [55]. All of these have two common main features, they were cleaved after dibasic sites or after single arginine and had a higher content of acidic amino acids (Asp and Glu). These were attributed to how neuroendocrine precursors are usually processed and their potential functional role as neurotransmitters by Glu-induced NMDA receptor activation [11,12,56]. In the following years, several authors have reported a few tens to one hundred peptides in CSF samples with precursor protein function predominantly associated with brain and neuronal function [41,42,43,57,58,59].

A comprehensive and in-depth characterization of the CSF peptidome significantly increased the number of identified peptides to 563 [44]. The following representatives of peptides and peptides families were identified: joining peptide, neurexophilins, synaptotagmins, FXYD, granulins, insulin-like growth factor-2, heparin-binding EGF-like growth factor, orexins, and convertase inhibitors (proSAAS and 7B2). It was predicted that the sequence characteristics of 84% of the precursor proteins resembled the sequence of prepro-hormones, indicating their signaling action, which was further supported by the fact that half of the precursor proteins of the detected peptides were not found in the proteome part of the sample, and thus, most likely, the identified peptides are not degradation products of proteins. Several post-translational modifications (PTMs) have been detected, such as sulfation, phosphorylation, and O-linked glycosylation.

Technological improvement on MS instrumentation over the years has enabled deepening the coverage of the CSF peptidome [46,60,61]. Combining a high sample amount (1.5 mL CSF) with sample pre-fractionation, state-of-the-art MS instruments, and using the advantages of the latest software development so far, the highest number of 18,031 peptide identifications was achieved [39]. These peptides originated from 2053 proteins, and more than 60 proteins have been linked to neurodegeneration. From the amyloid precursor protein, 213 peptides, 109 peptides from apolipoprotein E, and 6 peptides derived from microtubule-associated protein tau were identified.

## 5. CSF Peptidome in Alzheimer’s Disease

The first study applying the peptidomics approach for investigating AD biomarkers identified five peptide signals with altered abundance in the CSF samples of AD patients compared with healthy controls [62]. Four out of five detected peaks after purification with strong cation exchange chromatography and measured by MS were assigned to cystatin C, two β-2-microglobulin isoforms, and neurosecretory protein VGF (VGF). In a small sample cohort consisting of 9 AD patients and 10 healthy controls with no history, symptoms or signs of any psychiatric or neurological condition, the AD patients were distinguished from healthy donors with 100% sensitivity and 66% specificity. Thereby, a patient was classified as AD if the intensity of at least one of the up-regulated peaks was higher than the highest intensity found in the control group or if the intensity of the down-regulated peak was lower than the lowest intensity observed in the controls. Two years later, the same research team showed that specific cleavage of the N-terminal part of cystatin C is due to long-term storage at −20 °C, and the observed deregulation of its peptide does not reflect any pathological condition [63].

The very first study comprising a large number of subjects (*n* = 312) and investigating AD biomarkers, after extensive sample fractionation among 6000 detected MS signals identified 14 peptides derived from two proteins, one peptide from complement factor C3 (C3f) and the others were from VGF [64]. Eight VGF-derived peptides were significantly down-regulated in the CSF samples of AD patients compared with the non-dement controls. One VGF peptide (177–193) and C3f fragment distinguished AD from non-AD dementia. The activation of components of the complement system, i.e., C3b, in AD patients is induced by Aβ accumulation in the brain, thus triggering an inflammatory response by microglia activation aiming at their degradation [65]. The C3f peptide is released during the generation of iC3b from C3b [66]. Thus, a high C3f level might result from further processing of C3b to iCb3, with the consequence of reduced activation of the immune system. Therefore, up-regulation of C3f fragments in CSF was associated with the inactivation of the protective inflammatory response to the clearance of Aβ deposition, leading to the manifestation of AD pathology. VGF contains many dibasic sites in its sequence, i.e., adjacent arginine and/or lysine residues, which are specific cleavage sites of prohormone convertases [12]. Convertases can also cleave after only C-terminus arginine [12]. Seven out of nine down-regulated VGF-peptides are adjacent to either dibasic or monobasic amino acid residues, representing strong evidence of peptide generation by regulatory processes and most probably mediated by convertases. The controlled generation process and the potential regulatory function of down-regulated peptides were further supported by the presence of pyroglutamate residues at two peptides, which is a characteristic structural modification of neuropeptides [67]. The synthesis of VGF predominantly takes place in neurons and neuroendocrine cells [68,69]. Besides playing a critical role in many processes related to energy balance, synaptogenesis, neurogenesis, and memory, VGF peptides reduce Aβ plaque formation by triggering microglial activation through receptor binding [70]. Considering that VGF synthesis takes place in the neurons, the down-regulation of VGF peptides might indicate the disruption in the function of neurons and the progression of neuronal damage, which might have a consequence of down-scaling the protective mechanisms against Aβ deposition similar to the up-regulation of C3f, as discussed above.

The MS spectra of peptides isolated from postmortem-collected CSF samples with a two-stage peptide extraction protocol separated neuropathologically confirmed AD patients from non-demented controls in a PCA analysis [36]. It turned out that most discriminative peaks originated from VGF, complement C4 factor (C4), and alpha-2-HS-glycoprotein peptides. Again, VGF peptides showed a higher intense peak in the control and C4 in the AD samples. In contrast with the unmodified peptide of alpha-2-HS-glycoprotein and similar to the VGF peptide, the peak of the glycosylated form of alpha-2-HS-glycoprotein peptide showed a higher intensity in the control CSF samples. Although it is not so clear, the anti-inflammatory action mode has been attributed to alpha-2-HS-glycoprotein in AD patients and in the context of metabolic syndrome [71,72]. Therefore, down-regulation of its peptide and protein, also reported in the CSF and plasma of AD patients [72,73,74], might again indicate lowering of the protective mechanism, while up-regulation of C4 indicates the dominance of pro-inflammatory processes.

Significant down-regulation of 64 peptides in the AD group compared with the controls was also associated with neuronal degeneration and loss [38]. Here, the tandem mass tag (TMT) approach is implemented, which is a multiplex isobaric labeling strategy. As labeled standards are added to the sample before peptide extraction, the obtained data are more robust to experimental variations. Abundance reduction of several proteases (cathepsin B and carboxypeptidase) and protease inhibitors (plasma serine protease inhibitor, metalloproteinase 2 inhibitor) might interpret a general decrease in the peptide level. Peptides derived from the Bri domain of the integral membrane protein 2B (ITM2B) were identified for the first time [38]. A peptide from this domain has been reported to inhibit Aβ deposition in animal models [75].

The progress in MS technology and the development of powerful software packages enable identifying hundreds and thousands of peptides [39]. To keep a good overview of the data, despite the huge number of identified peptides, they are grouped into biological processes or pathways. So, the top biological categories in which the protein precursor of the 645 peptides identified in CSF samples execute their functions are cellular processes, biological regulation, metabolic processes, and organismal processes [76]. Secretory proteins made up the larger part with function distribution in binding, catalytic activity, and receptor activity. Peptides derived from proprotein convertase subtilisin/kexin type 1 inhibitor (proSAAS) and neurosecretory protein VGF had the highest number of representatives within many families of neuropeptides that were represented. From 42 proSAAS-derived peptides, the abundance of 80% of them decreased in the samples collected from AD patients, and the abundance level of two peptides (DHDVGSELPPEGVLGA and DHDVGSELPPEGVLG) fulfilling the applied quantification criteria decreased continuously from control, in mild cognitive impairment (MCI) from the control, and to AD patients. Surprisingly, all 42 proSAAS peptides were released upon either N- or C-terminal cleavage, which is a characteristic of proSAAS-driven bioactive peptides, such as big/little SAAS, PEN and LEN and hence indicating their possible physiological activity. Besides the identification of various fragments of SAAS, Pen, and LEN, the intact forms of LEN (SAAS245-260; LETPAPQVPARRLLPP) and little LEN (SAAS245-254; LETPAPQVPA) peptides were also discovered [76]. The reduction of proSAAS peptides in AD samples converged with the down-regulation of the proSAAS protein in AD patients [77], as well as the possible protective role on AD through its anti-aggregation effect on Aβ42 [78].

Capillary-electrophoresis coupled to mass spectrometry (CE-MS) analysis applied to CSF led to an AD diagnostic panel consisting of 12 signals, and only 5 of them were identified as following peptides or peptides fragments: ProSAAS precursor (217–242): AADHDVGSELPPEGVLGALLRV, chromogranin A (322–339): SGELEQEEERLSKEWEDS, phospholemman (21–41): ESPKEHDPFTYDYQSLQIGGL, clusterin (apolipoprotein-J) (22–49): DQTVSDNELQEMSNQGSKYVNKEIQNA, and VGF (26–59): GRPEAQPPPLSSEHKEPVAGDAVPGPKDGSAPEV [79]. The AD diagnostic pattern implemented in a prospective patient cohort to distinguish AD patients from the group of non-AD dementia and controls showed 87% sensitivity and 83% specificity, which was slightly better or comparable, in this study, to the diagnosis prediction-power obtained from measurement of beta-amyloid1–42, total-tau, and phospho181-tau with a sensitivity of 88% and specificity of 67%.

PTMs can be decisive in determining peptide function or its activation state. The high number of modified neuropeptides, 88 of the total 276 peptides listed in the NeuroPep database [53], is strong evidence of the impact of PTMs on brain function when considering the participation of neuropeptides in brain function-related processes [46]. PTMs attracted the attention of scientists from the beginning of peptidome analysis with CSF samples [36,44,60]. The fact that APP is glycosylated, the precursor protein of Aβ peptides, motivated scientists to investigate glycosylation forms of Aβ species leading to the identification of 64 Aβ glycopeptides, but none of them were from Aβ38, Aβ40, and Aβ42 isoforms [80]. In a patient cohort with only a few patients, a considerable up-regulation of Tyr10 glycosylated Aβ peptide fragments was concluded in AD patients compared with non-AD patients [80]. However, in a quantitative approach with 40 patients, the Aβ1-15 or Aβ1-17 glycopeptides were not found to be significantly regulated in AD patients [81].

The main challenge in the peptidomics analysis remains database search, which, through the study, of PTMs reaches another stage of complexity with search space expanding further. Nevertheless, a three-step database searching strategy and utilizing a softer dissociation technique (electron-transfer and higher-energy collision dissociation—EthcD) provided in-depth characterization of peptides glycosylation sites in CSF samples [46]. In total, 1411 peptide identifications were reported. Among them, 339 were post-translationally modified, of which 89 possessed glycosylation as PTMs. Most glycosylated forms were O-glycosylation, and only one peptide was N-glycosylated. The decrease in the number of identified peptides in the MCI and AD samples compared with the controls resulted from the reduction in the number of unmodified peptides rather than in the modified ones. On the contrary, within the identified peptides, an increasing trend in the percentage of O-glycosylation, Gln → pyro-Glu, acetylation, and phosphorylation of peptides in MCI and AD was observed. Only the percentage of oxidized peptides decreased. In the same study, 11 O-glycosylated LEN peptides and 3 O-glycosylated SAAS peptides, both originating from the ProSAAS protein, were identified.

An ideal biomarker would support the determination of a diagnosis in the best scenario at an early disease stage. A biomarker could also be suitable for monitoring disease progression or treatment response. In both cases, animal models represent a valuable option in the discovery part of biomarker research, but also in the understanding of the disease pathology and disease development. In CSF samples of a transgenic rat model for tauopathy and control animals from 345 identified peptides, those derived from 17 proteins showed abundance alteration: neurofilament light and medium chain, apolipoprotein E, gamma-synuclein, chromogranin A, reticulon-4, secretogranin-2, calsyntein-1 and -3, endothelin-3, neuroendocrine protein B72A, alpha-1-macroglobulin, fibrinogen beta chain, prosaposin receptor GPR37, receptor-type tyrosine-protein phosphatase zeta, small nuclear ribonucleoprotein-associated protein N and augurin [82]. Proteins enriched in the tau transgenic model are well known to mediate processes related to tau-induced neurodegeneration such as nervous system development, protein metabolic processes, or molecular processes such as peptidase and peptidase inhibitor activity, as well as receptor activity [82]. The altered proteins could serve as a starting point for further investigation in AD patients or conducting additional in vitro experiments, and in order to get new insights into AD pathogenesis, more effort needs to be spent on data analysis and data interpretation.

Attempts for monitoring of drug response administrated to humans are also known. Plaque formation from Aβ peptides associated predominantly with neuronal degeneration and cognitive decline is the central aspect of AD [83]. γ-secretase is one of the enzymes that, upon enzymatic cleavage of APP, guides the production of Aβ peptides [23]. Several treatment strategies have been implemented to inhibit enzymes implicated in Aβ production [84]. In an in-vivo study design, the potential of CSF was explored for the monitoring of the treatment response and the method of treatment action [61]. Semagacestat, a γ-secretase inhibitor, induced significant changes in human CSF samples, thus demonstrating the usefulness of peptidomics in drug treatment evaluation [61]. Among the 11 deregulated peptides, only two peptides derived from APP and one from amyloid precursor-like protein 1 are known to be directly linked to γ-secretase [61]. The changes of the other nine peptides might result from other disease-associated or unknown processes modulated by γ-secretase, pointing to the importance of implementing a global screening approach for investigating drug effects.

CSF is an ideal sample when processes related to brain and neuronal function are investigated. However, the collection of a CSF sample is more invasive compared with a routine clinical sample, such as of the plasma and serum. In this view, several studies have been conducted utilizing plasma and serum samples for biomarker discovery. So, the abundance of fibrinogen α chain (FIBA) derived peptide fibrinopeptide A (FPA, Aα1–16) and αC-domain (αCDC, Aα557–610), in combination with age, showed an area under the curve (AUC) of 0.717 in a small validation cohort of AD patients and controls [85]. The increase in intensity of both peptides in the serum of AD patients might describe the vascular damage in AD patients caused by atypical coagulation. By utilizing only 1.5 µL serum, a panel of 4 peptides (fibrinogen β chain, fibrinogen α chain, α2-HS-glycoprotein, and plasma protease C1 inhibitor) discriminated AD patients from controls with 87% sensitivity and 65% specificity, but only in a combination of all 4 peptides [86]. A study conducted with plasma samples showed up-regulation of peptides derived from common plasma proteins associated with innate immune response, such as proteins of the complement system, C2, C7, and C1QBP in AD patients compared with the controls [37].

## 6. Conclusions and Future Perspectives

Peptides in peptidomics experiments are much larger than those generated in conventional proteomics experiments after tryptic digestion [87]. As a consequence of this, they contain much higher charge states. Both represent a challenge in MS-based determination of the peptide sequence. Because of the enzymatic cleavage of basic amino acids in the process of generating a mature peptide, other peptides might not be charged at all and are thus not detectable. Otherwise, the immature form of the peptide is measured, which might not have significant biological meaning or its abundance might not change due to the enzymatic processing. The concentration of endogenous peptides in clinical samples compared with proteins, or after sample preparation compared with digested peptides is very low, making the peptidomics experiments highly exposed to any variation throughout the entire analysis. All of the factors mentioned above contribute to the poor reproducibility of peptidomics experiments. This becomes further complicated and is particularly important for biomarker research because the fold changes obtained in the peptidomics experiment are lower than those observed for proteins [38]. 

Another source of variation in peptidomics experiments is contamination with blood-derived peptides during CSF collection from patients. It might be a good strategy not to consider further interpreting the peptides identified in blood. However, there are known neuropeptides that act in organs other than the brain and that can also be found in the blood. Additionally, the presence of blood peptides in CSF might indicate a pathological condition such as impairment of the blood−CSF barrier. 

Although the normalization of data to the total identified proteins is widely spread in label-free proteomics experiments, it is questionable whether it is also suitable for peptidomics experiments considering the low number of identified peptides. Data normalization to the sample volume used for peptide extraction should be prioritized and is comparable with validation studies where targeted analysis is applied with volume-based data normalization.

Over the last two decades, the number of identified peptides in CSF increased tremendously. Achievement in MS technology has facilitated the determination of thousands of peptides in CSF. The introduction of the fourth dimension of peptide separation according to their mobility properties and, in addition to molecular weight separation at sample preparation, separation based on their hydrophobic properties in LC systems and mass to charge ratio in MS instrument, promises further deepening of the coverage of the CSF peptidome by MS-based analysis [88,89]. 

The main effort of the peptidomics community has been directed to AD, and all other neurodegenerative diseases are so far highly underrepresented, which is a potential to be explored in the future when considering the role of peptides in brain physiology. Although several hundreds of peptides derived from 42 proteins (Table 1) have been reported with an altered abundance in AD, the Aβ42 peptide still remains the prominent biomarker. With the exception of Aβ fragments, peptidomics data were interpreted in strong relation to the function of precursor proteins. In general, down-regulation of peptide abundance in AD has been attributed to neuronal dysfunction and loss associated with less peptide production. Abundance reduction in AD of peptides described to have a protective role against AD pathology indicates the down-scaling of protective mechanisms that might contribute to the occurrence of disease. On the contrary, an elevated level of peptides derived from proteins of the immune system has been explained as the immune response to a pathological condition. A higher identification number of glycosylated peptides illustrates the importance of PTMs in neurodegenerative disease, but also the maturation of the applied methodology. The potential of CSF and peptidomics analysis, not only in biomarker discovery, but also in monitoring disease progression and treatment response, is there and needs to be exploited.

## Figures and Tables

**Table 1:** Precursor proteins of peptides reported to be regulated in AD.

Protein Name	Gene Name	Regulated in AD	Reference
Alpha-2-HS-glycoprotein	AHSG	Down-regulated	[38,61]
Alpha-2-HS-glycoprotein	AHSG	Up-regulated	[36,86] *
Alpha-2-HS-glycoprotein (glycosylated)	AHSG	Down-regulated	[36]
Amyloid beta A4 protein	APP	Down-regulated	[38,61]
Amyloid-like protein 1	APLP1	Down-regulated	[38,61]
Beta-2-microglobulin	B2M	Up-regulated	[62]
Calsyntenin-1	CLSTN1	Down-regulated	[38]
CD99 antigen-like protein 2	CD99L2	Down-regulated	[61]
Chromogranin-A	CHGA	Up-regulated	[79] ***
Chromogranin-A	CHGA	Down-regulated	[38]
Clusterin (apolipoprotein-J)	CLU	Up-regulated	[79] ***
Complement C4 factor (C4)	C4A	Up-regulated	[36]
Complement component 1 Q subcomponent-binding protein, mitochondrial	C1QBP	Up-regulated	[37] **
Complement factor C2	C2	Up-regulated	[37] **
Complement factor C3 (C3f)	C3	Down-regulated	[64]
Complement factor C7	C7	Up-regulated	[37] **
Cystatin C	CST3	Up-regulated	[62]
Fibrinogen alpha chain	FGA	Down-regulated	[61]
Fibrinogen alpha chain	FGA	Up-regulated	[85,86] *
Fibrinogen beta chain	FGB	Down-regulated	[86] *
Golgi apparatus protein 1	GLG1	Down-regulated	[61]
Hyaluronan and proteoglycan link protein 2	HAPLN2	Down-regulated	[38]
Integral membrane protein 2B	ITM2B	Down-regulated	[38]
Integral membrane protein 2C	ITM2C	Down-regulated	[38]
Metallothionein-1E	MT1E	Down-regulated	[61]
Metallothionein-3	MT3	Down-regulated	[38]
Neuroendocrine convertase 2	PCSK2	Down-regulated	[38]
Neuromedin-S	NMS	Down-regulated	[38]
Neuron specific protein family member 1		Down-regulated	[38]
Neurosecretory protein VGF	VGF	Down-regulated	[36,38,62,64,79] ***
Phospholemman	FXYD1	Up-regulated	[79] ***
Plasma protease C1 inhibitor	SERPING1	Up-regulated	[86] *
Prepronociceptin	PNOC	Down-regulated	[38]
Proenkephalin-A	PENK	Down-regulated	[38]
ProSAAS	PCSK1N	Up-regulated	[79] ***
ProSAAS	PCSK1N	Down-regulated	[38,76]
Prosaposin receptor GPR37	GPR37	Up-regulated	[82]
Prostaglandin-H2 D-isomerase	PTGDS	Down-regulated	[38]
Secretogranin-1	CHGB	Down-regulated	[38]
Secretogranin-2	SCG2	Down-regulated	[38]
Secretogranin-3	SCG3	Down-regulated	[38]
Serum albumin	ALB	Down-regulated	[38]
Sodium/potassium/calcium exchanger 2	SLC24A2	Down-regulated	[38]
Sortilin	SORT1	Down-regulated	[38]
Superoxide dismutase	SOD1	Down-regulated	[38]
Tachykinin-3	TAC3	Down-regulated	[38,61]
Testican-1	SPOCK1	Down-regulated	[38,61]

* Serum. ** Plasma. *** CE-MS.

## Data Availability

Not applicable.
